# Transactions of the American Surgical Association

**Published:** 1889-10

**Authors:** 


### Book Reviews.

**Transactions of the American Surgical Association.** Volume VII. Edited by J. Ewing Mears, M. D., Recorder of the Association, with five illustrations. Cloth, 8vo, pp. xvi., 221. Philadelphia: Printed for the Association, and for sale by P. Blakiston, Son & Co. 1889.

The present volume sustains fully the reputation of the American Surgical Association for the good scientific work which it does. In variety and treatment of the papers presented, we doubt if any similar society can show a better record. Among the fifteen essays making up this volume, space permits mention of only a very few.

Dr. J. Collins Warren's paper on the Early Diagnosis of Malignant Growths, in which he advocates the use of a fine canula with sharp edges, and with which he removes a small portion of the tumor "en bloc," elicited a rather animated discussion as to the relative value of microscopical and clinical evidence in the diagnosis of malignant growths. The majority of the surgeons discussing the paper seemed to place rather small confidence in the findings of the microscope, and would only rely upon them when corroborated by the clinical aspects of each individual case.

The essay entitled Hernia: a Comparison of the Various Methods Adopted for its Cure, by Dr. Claudius H. Mastin, is an admirable review of this all-important subject. The author begins his historical rêsumê with the methods of Celsus, viz.: Incision, castration, cauterization, and bandage, and mentions in turn cauterization by acids and moxas, topical applications, as poultices and plasters, ligature of the sac, the royal suture, the scarifications of Le Blanc, invagination, without and with suture. Then he considers the various methods of later times, such as bruises, injections, subcutaneous operations, and the many modifications of the open treatment. Czerny receives the credit of introducing the last mentioned method, which the author briefly but fully describes. After discussing the modes of operating pursued by Ball, Macewen, Banks, Stokes, Alexander, McBurney, and others, Dr. Mastin comes to the following conclusion regarding indications for and methods of operating, which is best given in his own words:

From a comparison of all the methods, it is apparent that no fixed rule or procedure is established, and, although the radical operation is a marked improvement in the treatment of hernia, whether free or strangulated, we cannot consider it perfected, because the methods hitherto resorted to have not proved radical in results. The operation is ideally correct, but the question arises whether, with the uncertainty of success, the risk justifies the operation; especially so, if the circumstances of the individual are such that he can content himself with the use of a properly adjusted truss.

The discussion which followed is interesting and valuable. Each surgeon prefers this or that method, but all manifest a marked degree of conservatism in the cases which are deemed suited for operation. Dr. Agnew would limit the radical cure to strangulated and rebellious hernias, which cannot be controlled by a truss, and which endanger the lives of the patients, and in this opinion his colleagues mostly concur. Whoever wishes to get a good knowledge of the history of hernia, a careful discussion of the various indications for and against its radical cure, and of the many methods of obtaining the same, cannot do better than read this masterly paper of Dr. Mastin.

Another excellent paper is that upon Gunshot Wounds of the Intestines, by Dr. Theodore A. McGraw. The propositions which it advances are so original and striking that we feel justified in devoting a little space to it. The author has stated his views in the form of nine propositions, which may be briefly epitomized as follows:

*Proposition First.*—The gravity of an injury of this kind (gunshot wound of the intestines) depends partly upon the size of the missile. Experiments upon animals showed that extravasation was less likely to follow wounds from missiles of small than of large calibre.

*Proposition Second.*—Bullets which enter the abdominal cavity pass in a nearly absolutely straight line from the orifice of entrance through the peritoneum to that of exit; or, to their final stopping place in the viscera. Sixteen bullets were fired through the bodies of dead animals, only one showing any deviation from a perfectly straight line, and this but two centimeters in a course twenty-five centimeters long. Of nine bullets fired through living sheep, all went straight except one, which was lost. These experiments lead the author to believe that the idea of bullets glancing within the abdominal cavity is an incorrect one, and that "almost all apparent deflections of bullets fired into the abdomen are due to subsequent changes in the position of the abdominal walls or viscera, caused either by muscular contraction and relaxation, or by a change in the position of the intestines from the passage of gas and other causes."

*Proposition Third.*—An incision made directly in the course of the ball will give the shortest route to the injured parts. It has, beside, all the advantages of the median incision.

*Proposition Fourth.*—The contents of the bowel may be made to discharge through an open gunshot wound by manipulation and pressure. If no discharge occurs, it is because the wound has been closed, either by eversion of membrane or by exudation of plastic lymph, and, in either case, will probably recover without suture if aseptic and the bowels quiet.

*Proposition Fifth.*—An empty condition of the alimentary canal is most favorable to healing. To secure this, evacuate the stomach when necessary. In small wounds of the stomach and duodenum, suture may be sometimes omitted if those viscera are empty.

*Proposition Sixth.*—Agglutination and limitation of the morbid process, consequent upon gunshot wounds of the intestines, may take place as early as the sixth day (hour ?). Keep the abdominal incision entirely within the limits of the morbid area. We should then treat by irrigation and drainage, and postpone all further operative procedures until inflammatory action has all passed. All wounds of the abdomen, more than six hours old, should be incised in the line of the ball.

*Proposition Seventh.*—Senns' method of hydrogen-gas insufflation, however admirable in recent cases, should be used with great caution after the lapse of a few hours. It can cause rupture of inflammatory adhesions, burst open intestinal wounds nearly healed, and make a circumscribed peritonitis a general one.

*Proposition Eighth.*—The dangers of an operation for penetrating gunshot wounds of the abdomen are directly in proportion to the length of the same, and to the amount of evisceration. The length of an operation may be lessened by (1) examining only the viscera in the course of the ball; (2) suturing wounds, when possible, instead of excising them; (3) omitting all operative procedures in all wounds so thoroughly occluded by plastic material, that the contents of the bowel cannot pass through them; (4) operating first on those wounds which imperatively demand it (for instance, when stomach and small intestine are both perforated, attend to the latter first, because the empty stomach may recover from large wounds without suture), and suturing large before small wounds, and wounds discharging before those which are occluded; (5) never eviscerating a patient except for hemorrhage otherwise uncontrollable, or to find a discharging wound otherwise undiscoverable. The author is most positive in his conviction that evisceration, and even slipping the intestine through the fingers from one end to the other, are never to be done as routine practice. Both procedures cause great shock to patients usually exhausted. He says "it is better to have an occasional patient die of an undiscovered wound rather than to kill many by unnecessary procedures." A careful study of the course of the ball will render manipulation unnecessary of any viscera, or portions of intestine lying without the immediate neighborhood of the path of the missile.

*Proposition Ninth.*—In cases in which the patients are too weak to endure the radical operations (laparatomy and repair of intestinal wounds), laparatomy and drainage should take their place.

The paper caused a spirited discussion, in which the author's conclusions were greatly criticised. Most of the Fellows who entered into the discussion took opposite views concerning the straight course invariably taken by the missile, they believing that deflection does occur. They also advocate the median incision and evisceration, or at least thorough examination of the entire intestinal tract in all cases, rather than following merely the path of the ball.

There are several other papers in this volume which deserve extended notice, but which cannot be here given. All in all, the transactions for 1889 make an interesting and valuable volume, which the surgeon will do well to carefully read. J. P.

